# A Scoping Review of Psychological Sense of Community among Community Dwelling Older Adults

**DOI:** 10.1093/geroni/igab046.3454

**Published:** 2021-12-17

**Authors:** Thomas Buckley

**Affiliations:** University of Pittsburgh, Virginia Beach, Virginia, United States

## Abstract

Psychological sense of community (PSOC) is an important construct for health and well-being outcomes for older adults. Drawing on the Ecological Theory of Aging and the Age-Friendly Cities (AFC) framework, this scoping review explored how PSOC has been used in research with community dwelling older adults. I followed Arksey and O’Malley's (2005) scoping review guidelines. Initial database searches yielded 860 articles. I included 33 in the final sample. I grouped articles based on study populations and conceptualization and operationalization of PSOC. I used thematic analysis to explore topic areas and main findings. The AFC framework guided development of themes and others emerged during analysis. Results show most studies used Asian or White samples and focused on geographic community or neighborhoods. Among the several measures of PSOC, the Brief Sense of Community Scale performed best with older adults. Topical research areas in the thematic analysis were built (1) built environment and neighborhoods, (2) social participation and connection, (3) civic participation, (4) PSOC as a protective factor, (5) health and well-being, (6) relocation, and (7) scale development. PSOC was a consistent predictor of health and well-being and served as a mediator to link neighborhood or environmental characteristics with health and well-being. Future research needs to examine PSOC in geographically and culturally diverse samples and conduct further psychometric testing of PSOC scales with older adults. PSOC is conceptually related to the AFC framework and serves as a mechanism that links AFC features and well-being outcomes. These results can inform practice and refine theory.

